# Adenosine receptor agonist NECA increases cerebral extravasation of fluorescein and low molecular weight dextran independent of blood-brain barrier modulation

**DOI:** 10.1038/srep23882

**Published:** 2016-03-30

**Authors:** Chih-Chung Cheng, Ya Lan Yang, Kate Hsiurong Liao, Ted Weita Lai

**Affiliations:** 1 Graduate Institute of Clinical Medical Science, China Medical University, Taichung 404, Taiwan; 2 Translational Medicine Research Center, China Medical University Hospital, Taichung 404, Taiwan

## Abstract

Conventional methods for therapeutic blood-brain barrier (BBB) disruption facilitate drug delivery but are cumbersome to perform. A previous study demonstrated that adenosine receptor (AR) stimulation by 5′-N-ethylcarboxamide adenosine (NECA) increased the extravasation of intravascular tracers into the brain and proposed that AR agonism may be an effective method for therapeutic BBB disruption. We attempted to confirm the extravasation of tracers into the brain and also investigated tracer extravasation into peripheral organs and tracer retention in the blood. We found that NECA not only increased the extravasation of intravascular fluorescein and low molecular weight dextran into the brain of mice but also increased the concentrations of these tracers in the blood. In fact, the brain:blood ratio-normalized BBB permeability for either tracer is actually decreased by NECA administration. Elevated blood urea nitrogen levels in mice following NECA treatment suggested that renal function impairment was a probable cause of tracer retention. Therefore, NECA has almost no effect on the extravasation of intravascular Evans blue dye (EBD), an albumin-binding tracer with little renal clearance. Rather than inducing BBB disruption, our study demonstrated that NECA increased tracer extravasation into the brain by increasing the concentration gradient of the tracer across the BBB.

The blood-brain barrier (BBB) not only protects the brain against unwanted exchanges of chemicals and proteins but also impedes therapeutic agents from entering the brain. Methods to therapeutically disrupt the BBB have been developed to facilitate drug delivery^1^,^2^,^3^,^4^, and their use has been shown to increase the efficacy of chemotherapy against brain tumors in experimental studies^5^,^6^,^7^,^8^,^9^,^10^ and in clinical trials^11^,^12^,^13^. However, these methods are technically demanding and can cause serious adverse effects^14^,^15^,^16^,^17^,^18^.

Adenosine receptors (ARs) have recently been regarded as promising pharmacological targets for inducing BBB disruption^19^. This notion is based on a study showing that the intravenous administration of 5′-N-ethylcarboxamide adenosine (NECA), a broad spectrum AR agonist, increases brain extravasation of intravascular low molecular weight (LMW) and high molecular weight (HMW) dextrans^20^. Interestingly, this pharmacological effect of NECA was somewhat dose-specific, with a maximal effect at 0.08 mg/kg but sub-maximal effects at either lower doses of 0.02 and 0.04 mg/kg or higher doses of 0.16 and 0.32 mg/kg^20^. It was postulated that NECA at doses above 0.08 mg/kg desensitized the ARs, leading to reduced pharmacological responses. Consistent with AR signaling, the efficacy by which NECA induces brain extravasation of intravascular tracers was attenuated in mice lacking either A_1_ or A_2A_ receptor subtypes and in mice receiving antagonists against the ARs^20^. These findings could be of tremendous translational value because AR agonists are already clinically approved for other indications, and the intravenous administration of NECA is far less invasive than existing methods for disrupting the BBB.

The discovery that AR agonism may disrupt the BBB is surprising because poor BBB permeability was reportedly a primary obstacle in the development of AR agonists as drug candidates for treating central disorders^21^,^22^. For instance, AR agonists cause potent vasodilatation when applied centrally, but often has no effect on cerebral blood flow when administered intravenously^21^,^23^. In addition, *in vitro* studies have yielded controversial results. Although AR agonists decreased trans-endothelial electrical resistance (TEER), which measures paracellular resistance, across an endothelial monolayer^20^,^24^,^25^, it had no effect on TEER or tracer permeability when the endothelial monolayer was co-cultured with astrocytes^22^, which mimics the BBB inducing microenvironment of the brain^26^,^27^. The discrepancy between that *in vitro* study^22^ and the recent *in vivo* findings^20^ suggests that the increased extravasation of intravascular tracer, as a measure of therapeutic BBB disruption, induced by NECA and other AR agonists did not result from a direct effect on the BBB.

The original aim of this study was to evaluate the efficacy of NECA in inducing BBB disruption to facilitate the transport of neuroprotective agents^28^ into the brain. However, we found that NECA strongly increased the plasma concentrations of inert tracers commonly used to measure BBB permeability. Thus, rather than acting on the BBB directly, NECA indirectly increased the extravasation of these tracers across the BBB by increasing their concentration gradients.

## Results

### NECA increases brain extravasation of fluorescein without BBB disruption

**Figure 1:**
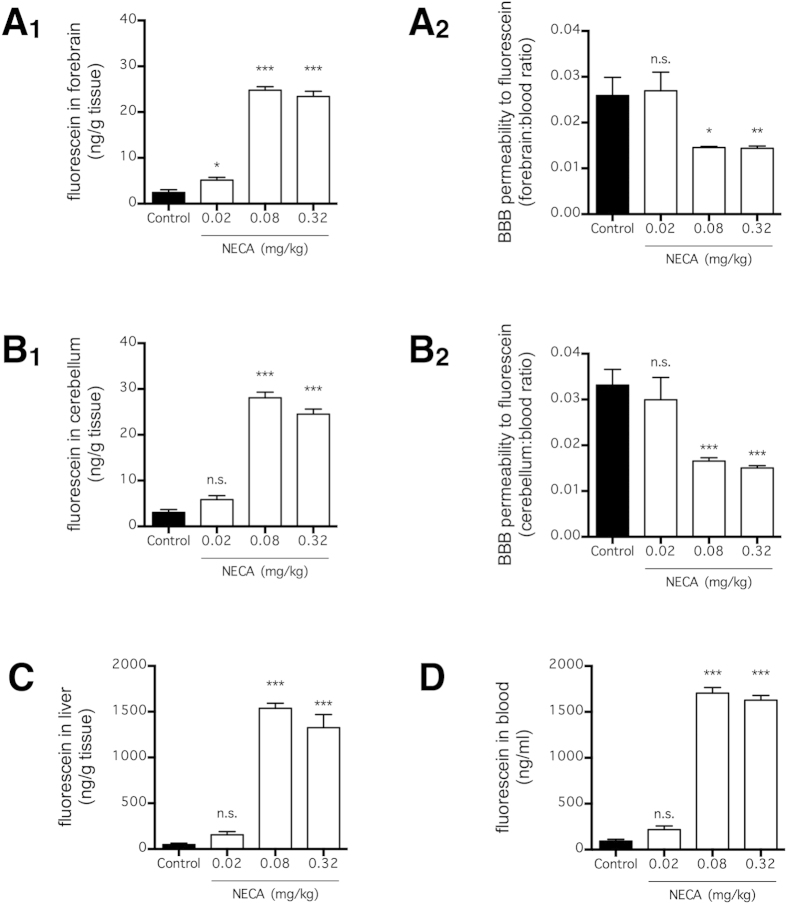
NECA increased fluorescein extravasation and concentration in blood. Mice received fluorescein (i.v. via tail vein) at 3 h after NECA injection (n = 6 per group), and fluorescein concentrations in forebrain (**A**_**1**_), cerebellum (**B**_**1**_), liver (**C**), and blood (**D**) were quantified 90 min later. BBB permeability was normalized to fluorescein concentration in blood for fluorescein concentrations in forebrain (**A**_**2**_) and cerebellum (**B**_**2**_). The bars represent means ± SEM. Significant differences were determined by Fisher’s LSD test. *p < 0.05, **p < 0.01, and ***p < 0.001; n.s. indicates no significant difference.

To determine BBB permeability, mice were injected with fluorescein, a LMW tracer that does not normally cross the BBB^29^, 3 h after NECA administration (Fig. 1). Thereafter, the amount of fluorescein that extravasated into the brain parenchyma was quantified (Fig. 1A,B). The dose and route of administration for NECA and the time point for assessment of BBB permeability were based on the original study that demonstrated the BBB disrupting effect of AR stimulation^20^. As discovered in that study, NECA dose-dependently increased the amount of fluorescein extravasated into the forebrain and cerebellum, with 0.08 mg/kg being the most effective dose (p < 0.001; n = 6) (Fig. 1A,B). However, when fluorescein levels in the liver and blood were measured, we also observed a substantial increase in fluorescein concentration (p < 0.001; n = 6) (Fig. 1C,D). Additionally, higher doses of NECA (0.08 and 0.32 mg/kg) actually decreased the normalized BBB permeability (p < 0.001; n = 6), which was calculated by taking the ratio of brain:blood fluorescein concentrations (Fig. 1A,B). These findings suggest that NECA increased the brain extravasation of fluorescein by increasing its plasma concentration, but the effect on fluorescein extravasation was less effective at higher doses (>0.08 mg/kg) due to a gradual decrease in BBB permeability.

### NECA increases brain extravasation of LMW dextran without BBB disruption

**Figure 2:**
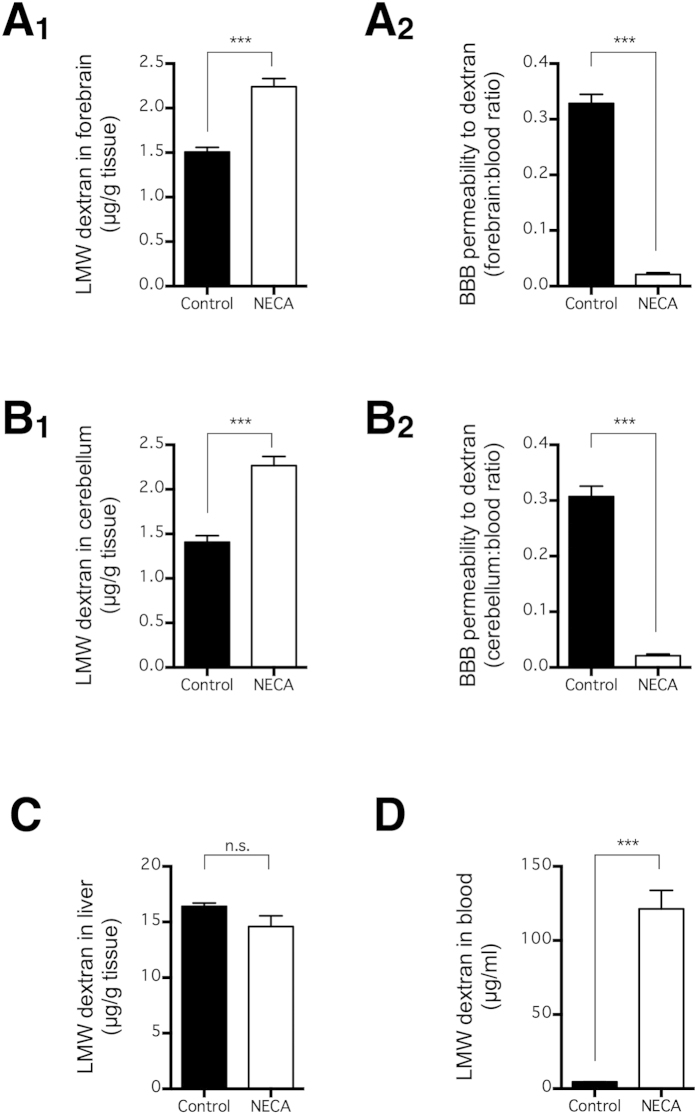
NECA increased low molecular weight (LMW) dextran extravasation and concentration in blood. Mice received LMW dextran (i.v. via tail vein) at 3 h after NECA injection (n = 10 per group), and LMW dextran concentrations in forebrain (**A**_**1**_), cerebellum (**B**_**1**_), liver (**C**), and blood (**D**) were quantified 90 min later. BBB permeability was normalized to LMW dextran concentration in blood for LMW dextran concentrations in forebrain (**A**_**2**_) and cerebellum (**B**_**2**_). The bars represent means ± SEM. Significant differences were determined by the unpaired t test. ***p < 0.001; n.s. indicates no significant difference.

To examine whether our unexpected findings were specific to fluorescein, we repeated the same experiment using LMW dextran, the same tracer used in the original study^20^ that concluded that NECA opens the BBB. Consistent with the above findings, NECA (0.08 mg/kg) significantly increased the amount of LMW dextran extravasated into the forebrain and cerebellum (p < 0.001; n = 10) (Fig. 2A,B), but it also, even more substantially, increased the concentration of LMW dextran in blood (p < 0.001; n = 10) (Fig. 2D). Therefore, NECA decreased the normalized BBB permeability calculated by taking the ratio of brain:blood LMW dextran concentrations (p < 0.001; n = 10) (Fig. 2A,B). Taken together, NECA increased the brain extravasation of LMW fluorescein and dextran by increasing their plasma concentrations; this was despite a decrease in BBB permeability, determined by normalizing the differences in plasma tracer concentrations.

### Increase in tracer concentration is associated with impaired renal function

**Figure 3:**
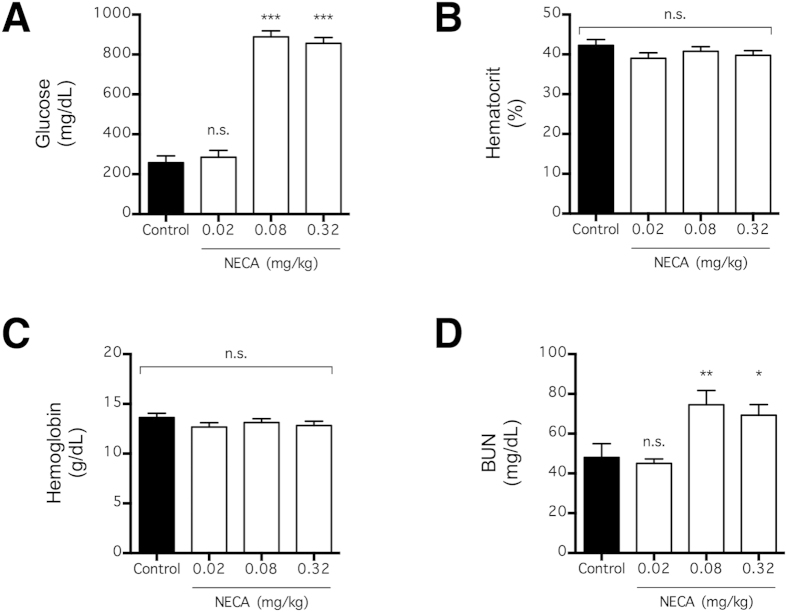
Systemic effect of NECA on glucose and renal function. Blood glucose (**A**), hematocrit (**B**), blood hemoglobin (**C**), and blood urea nitrogen (BUN) (**D**) were determined 4.5 h following NECA injection in mice (n = 4 per group). The bars represent means ± SEM. Significant differences are determined by Fisher’s LSD test. *p < 0.05, **p < 0.01, and ***p < 0.001; n.s. indicates no significant difference.

Given that the plasma concentration of intravascular tracer was increased, we examined the blood of NECA-treated mice to understand this pharmacological response (Fig. 3). Consistent with the AR-dependent effect of NECA on blood glucose levels in mice^30^, NECA (0.08 and 0.32 mg/kg) increased the plasma glucose concentration (p < 0.001; n = 4) in mice used in this study (Fig. 3A). This confirmed that our protocol for preparing and injecting NECA was technically competent.

Because AR signaling is known to affect urine volume^31^,^32^, the increased plasma fluorescein concentration could be secondary to a change in blood volume. In this experiment, NECA (0.02–0.32 mg/kg) failed to have any effect on the hematocrit or hemoglobin concentration, two indicators of acute change in blood volume, of the treated mice at 4.5 h following intravenous injection (p > 0.05; n = 4) (Fig. 3B,C). Thus, any effect of NECA on blood volume is unlikely to affect plasma fluorescein concentration in the time course of this study.

The previous study reported that NECA-induced BBB leakage was substantially attenuated in A_1_-knockout mice^20^. Interestingly, AR signaling via the A_1_ receptor subtype is also known to contribute to acute renal failure induced by cytotoxic agents^33^,^34^,^35^. Consistent with acute renal impairment, mice that received NECA (0.08 and 0.32 mg/kg) in this study showed increased BUN (p < 0.001; n = 4), an indicator of renal deficit, within the time course of the experiments (Fig. 3D). Therefore, the NECA-induced acute increase in plasma concentrations of the tracers (Figs 1D and 2D), which increased their extravasation into the brain (Figs 1A,B and 2A,B) indicating a perceptual disruption of the BBB, could result from the decreased renal excretion of these tracers.

**Figure 4:**
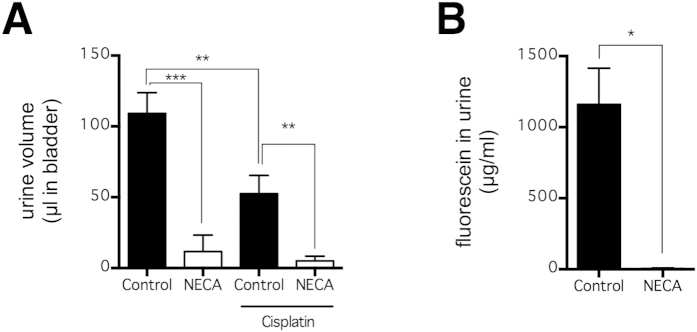
NECA decreased urine volume and fluorescein excretion. Mice received fluorescein (i.v. via tail vein) at 3 h after NECA injection (n = 6 per group), and urine volume (**A**) and fluorescein concentration (**B**) were quantified 90 min later. A subset of mice also received cisplatin, a chemotherapy agent known to induce kidney injury, 3 d prior to the experiment. The bars represent means ± SEM. Significant differences are determined by Fisher’s LSD test. *p < 0.05, **p < 0.01, and ***p < 0.001; n.s. indicates no significant difference.

To directly assess whether NECA can reduce urine volume and renal clearance of fluorescein, mice again received NECA (0.08 mg/kg) and fluorescein (3 h later), and this time the volume and fluorescein concentration of their urine remaining in bladder 4.5 h after NECA injection were determined (Fig. 4). As a mild positive control, cisplatin (16 mg/kg), a chemotherapeutic agent that impairs kidney function, was injected in a subset of mice 3 days prior to the experiment. Cisplatin moderately decreased urine volume (p < 0.01; n = 6) (Fig. 4A), but had no effect on fluorescein concentration in urine (p > 0.05; n = 6). In comparison, NECA is much more effective in impairing kidney function; it substantially decreased urine volume in either control mice (p < 0.001; n = 6) or cisplatin-treated mice (p < 0.01; n = 6) (Fig. 4A). In addition, NECA also substantially decreased fluorescein concentration in urine (p < 0.05; n = 3–12) (Fig. 4B). Notably, the concentration of fluorescein is much higher in urine (Fig. 4B) than it is in the blood (Fig. 1D), suggesting that renal clearance is a primary route of fluorescein excretion. Taken together, our data demonstrates that NECA increased fluorescein in blood by impairing renal function.

### NECA-induced tracer extravasation is attenuated when tracer is bound to plasma albumin

**Figure 5:**
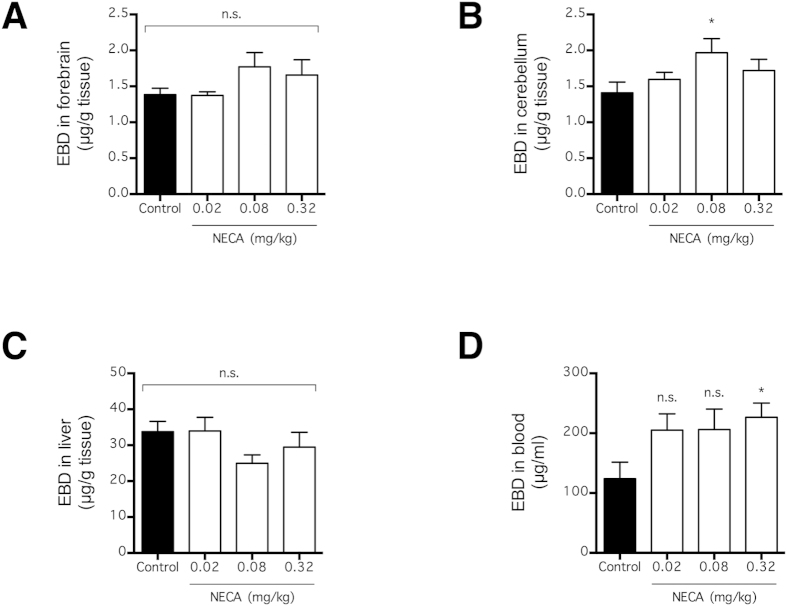
NECA has little effect on EBD extravasation and concentration in blood. Mice received EBD (i.v. via tail vein) at 3 h after NECA injection (n = 10 per group), and EBD concentrations in forebrain (**A**), cerebellum (**B**), liver (**C**), and blood (**D**) were quantified 90 min later. The bars represent means ± SEM. Significant differences are determined by Fisher’s LSD test. *p < 0.05; n.s. indicates no significant difference.

Our data were based on the systemic distribution of intravenous tracers and suggests that NECA increased the extravasation of intravascular tracers by preventing their renal clearance from blood (Figs 1 and 2). Therefore, NECA is expected to be less effective at inducing the extravasation of a tracer that is retained in the blood regardless of renal performance. To test this possibility, mice were injected with the Evans blue dye (EBD), a tracer that binds strongly to plasma albumin and hence, exhibits more plasma retention than an equivalent dose of fluorescein^29^, 3 h after NECA administration (Fig. 5). As with fluorescein and LMW dextran, the amounts of EBD that extravasated into the brain and liver parenchyma were quantified 90 min following tracer injection. Consistent with our hypothesis, the NECA-induced tracer extravasation into the brain and the liver was strongly attenuated (Fig. 5A–C). In fact, there was no significant increase in EBD extravasation into the forebrain or the liver (p > 0.05; n = 10) (Fig. 5A,C), and only a moderate significant increase in EBD extravasation into the cerebellum (p < 0.05; n = 10) (Fig. 5B). NECA also moderately increased the plasma EBD concentration (p < 0.05 for 0.32 mg/kg; n = 10) (Fig. 5D), but not as much as the plasma fluorescein and LMW dextran concentrations (Figs 1D and 2D). These observations agreed with our data suggesting that NECA increased the tracers’ plasma concentrations by impairing their clearance rather than changing plasma volume (Fig. 3). Because NECA was less effective at facilitating the extravasation of EBD into the central and peripheral tissues (Fig. 5A–C), its effect on plasma tracer concentration is likely crucial for its effect on tracer extravasation across the BBB.

## Discussion

Recent progress in understanding the cellular mechanism of neuronal death led to the development of compounds that are effective in animal models of neurodegeneration^28^. Given that several of these compounds have poor BBB permeability, their translational value can be greatly augmented with a safe and reliable method for disrupting the BBB. Existing methods to disrupt the BBB include the intracarotid administration of hyperosmolar solutions^1^,^2^ and ultrasound stimulation^3^,^4^. The former requires extensive surgery to cannulate the external carotid artery, and the latter method requires expensive equipment that is not widely available. In addition, these methods carry risks for serious adverse effects^14^,^15^,^16^,^17^,^18^. Based on the findings that NECA increased the extravasation of intravascular tracers into the brain^20^, AR agonism is widely regarded to be a feasible strategy for disrupting the BBB^19^,^36^. Unlike intracarotid administration of hyperosmolar solutions and ultrasound stimulation, AR agonism requires no invasive surgery, no expensive equipment, and can be formulated with neuroprotective agents.

We aimed to take advantage of AR agonism in the delivery of neuroprotective agents into the brain in our animal models of neurodegenerative diseases. Unexpectedly, however, we found that AR agonism by NECA, the prototypical broad-spectrum AR agonist best characterized for BBB disruption^20^,^36^, did not increase BBB permeability for fluorescein (Fig. 1A,B) and LMW dextran (Fig. 2A,B). Rather, NECA increased the extravasation of these intravascular tracers into the brain by increasing the plasma concentrations of the tracer (Figs 1D and 2D). This result was in marked comparison to BBB disruption by an intra-carotid infusion of hyperosmolar mannitol, which has been shown to increase tracer extravasation into the brain without affecting tracer concentration in the blood^37^. Unfortunately, the previous studies that investigated the effect of AR agonism on BBB disruption only measured tracer concentration in the brain parenchyma and not that in the blood^20^,^24^,^36^.

Consistent with the data reported by Carman *et al*.^20^, we found that NECA was most effective at inducing tracer extravasation at a dose of 0.08 mg/kg, compared with lower or higher doses (Figs 1 and 5). To explain this peculiar dose-response relationship, Carman *et al*. postulated that higher doses of NECA were less effective due to a desensitization of ARs^20^. In the present study, when the BBB permeability was normalized based on the brain:blood ratio of the tracer, NECA appeared to have no effect on BBB permeability at a lower dose (0.02 mg/kg) (Fig. 1A,B) and substantially decreased the permeability at higher doses (0.08 and 0.32 mg/kg) (Fig. 1A,B). In contrast to the observed BBB permeability trend, a NECA-induced increase in the plasma tracer concentration was most effective at 0.08 mg/kg (Fig. 1D). Based on our data, we proposed that NECA increased tracer extravasation at lower doses (<0.08 mg/kg) by increasing plasma tracer concentration, but decreased tracer extravasation at higher doses (>0.08 mg/kg) by a distinct mechanism that decreased BBB permeability.

A plausible mechanism by which NECA decreased extravasation of tracers across the BBB is by increasing the expression of transporters that exports these tracers out of the brain. For instance, the multidrug resistance-associated proteins (MRPs) are transporters expressed in cerebral endothelial cells that selectively binds to anionic molecules, including fluorescein^38^,^39^,^40^. Although genetic knockout of MRP-1, a functional MRP subtype expressed in cerebral endothelial cells, in mice has no effect on BBB permeability of fluorescein^41^, general inhibition of MRPs by a number of inhibitors increased extravasation of fluorescein into the mouse brain^41^,^42^. Indeed, MRP-mediated transport of fluorescein is increased by direct glucose application^43^, and this partially explains why BBB permeability to fluorescein is poor in diabetic animals^44^. Given that NECA substantially increased blood glucose concentration (Fig. 3A), it is possible that glucose-mediated increased in MRP expression contributed to decreased BBB permeability to fluorescein (Fig. 1A,B). In any case, given that NECA was administered systemically and that it stimulates all known AR subtypes, the net effect of NECA was likely multifactorial.

In conclusion, the two most widely used methods for disrupting the BBB, the osmotic disruption of BBB by intra-carotid hyperosmolar mannitol and microbubble-assisted focused ultrasound, have been shown to be effective in the brain delivery of therapeutic compounds. However, both methods are technically demanding and associated with serious adverse effects when performed incompetently. Despite our failed attempt at using AR agonism for BBB disruption, we continue to be hopeful for the development of new methods of BBB disruption that have the ease of AR agonism and without the adverse effects associated with existing methods.

## Methods

### Mice and *in vivo* procedures

Male C57BL/6 mice (21–27 g) were used in this study, and experiments were carried out in accordance to the Institutional Guidelines of the China Medical University for the Care and Use of Experimental Animals (IGCMU-CUEA) and were approved by the Institutional Animal Care and Use Committee (IACUC) of China Medical University (Taichung, Taiwan) (Protocol No. 103-224-NH). The mice had free access to rodent chow and water prior to the experiment. On the day of the experiment, mice were briefly anesthetized by isoflurane (2%, carried by air) and received either vehicle (10 ml/kg, 0.3% DMSO in saline) or NECA (0.02–0.32 mg/kg) via tail vein injection. At 3 h following NECA injection, each mouse was briefly re-anesthetized to receive a bolus injection of either tracer (i.v. through tail vein), sodium fluorescein (4% solution in saline; 2 ml/kg), LMW (10 kDa; FITC-labeled) dextran (10 mg/ml solution in PBS; 4 ml/kg), or EBD (4% solution in saline; 2 ml/kg). After another 90-min period to allow for tracer extravasation (or 4.5 h following NECA injection), the mice were euthanized by an overdose of urethane. Their dye-stained blood were collected from the heart and immediately placed on ice to slow down hemolysis. The mice were then perfused thoroughly with saline to remove residual blood from the vascular lumen, and the perfused brain and liver tissues were collected. In all experiments, NECA was prepared and randomly coded by a research assistant and blinded to the investigators until the end of all data collection.

### Tissue processing and tracer quantitation

Extraction and quantitation of the tracers were carried out using a recently reported method^45^. Briefly, brain and liver tissues were homogenized for 10 min in 1:3 ratios (wt:vol) with 50% TCA, and the tissue debris and protein precipitates were removed by centrifugation at 10,000 × g for 20 min. The blood samples were mixed in 1:3 volume ratios of 50% TCA, and centrifuged to remove protein and cellular precipitates. For the LMW dextrans, the TCA solution was pH adjusted to 7–8. The extracted dye from brain, liver, and blood were added to a clear 96-well plate at 30 μl per well. Each well was supplemented with 90 μl of 95% ethanol to intensify fluorescent signal^45^, and the dye concentrations were quantified by spectroscopy. In a subset of mice that did not receive an intravascular tracer, blood samples were collected 4.5 h following NECA injection and directly subjected to analysis by a blood electrolyte analyzer (Stat Profile Critical Care Xpress, Nova Biomedical) to determine the concentrations of blood glucose, hematocrit, hemoglobin, blood urea nitrogen (BUN), and elemental electrolytes.

### Urine volume and fluorescein concentration

Mice received NECA and sodium fluorescein as described earlier, and urine was collected from the bladder thereafter (4.5 h after NECA). Kidney injury was induced in a subset of mice by cisplatin (16 mg/kg, i.p.) pre-treated 3 d prior to the experiment. The volume of urine was measured using a syringe, and fluorescein concentration was quantified. Because only 3 out of 12 NECA-treated mice had sufficient urine for fluorescein quantification, comparison of fluorescein concentration was made between 12 control mice and 3 NECA-treated mice.

### Data presentation and analysis

Data were presented as means ± SEM. Significant differences between treatment groups were determined by the unpaired t test or the Fisher’s least significant difference (LSD) test.

## Additional Information

**How to cite this article**: Cheng, C.-C. *et al*. Adenosine receptor agonist NECA increases cerebral extravasation of fluorescein and low molecular weight dextran independent of blood-brain barrier modulation. *Sci. Rep*. **6**, 23882; doi: 10.1038/srep23882 (2016).
